# Measurement of Electrokinetically induced hydrodynamics at Ion-selective interfaces using 3D Micro particle tracking velocimetry (µPTV)

**DOI:** 10.1016/j.mex.2022.101814

**Published:** 2022-08-08

**Authors:** Felix Stockmeier, Michael Schatz, Malte Habermann, John Linkhorst, Ali Mani, Matthias Wessling

**Affiliations:** a Chemical Process Engineering AVT.CVT, RWTH Aachen University, Germany; b DWI - Leibniz-Institute for Interactive Materials, Germany; c Department of Mechanical Engineering, Stanford University, Germany

**Keywords:** Electroconvection, 3D Velocity Field, Ion-Exchange Membrane

## Abstract

Electrokinetic flow phenomena are ubiquitous in electrical systems for desalination, chemical conversion, or mixing at a micro-scale. However, the important features of resulting 3D flow fields are only accessible through cost-intensive numerical simulations. Experimental 2D recording of the chaotic three-dimensional velocity fields developing for example at currents exceeding the limiting current density does not capture the complex 3D structures present in such flow fields. Additionally, numerical 3D studies are limited to dimensions three orders of magnitude smaller as found in real applications and only short run times due to the enormous computational effort. To apply the theoretical knowledge in real-world systems and create the possibility for detailed parameter studies, we present the first experimental method for recording and quantifying the time-resolved velocity field in an electrochemical microfluidic cell in 3D with dimensions found in industrial applications. We utilize this method in a co-submitted paper to record the 3D velocity field of electroconvection at a cation-exchange membrane.

• Cell design suitable for simultaneous electrochemical experiments with optical 3D velocity quantification
• Method optimized for velocity reconstruction of membrane-to-membrane distances found in industrial cells
• Highly adaptable cell design, for optical characterization of electrochemical systems

## Graphical abstract

**Figure:**
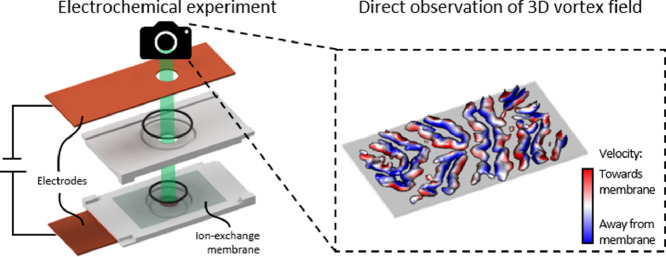

Specifications table

**Table:** 

Subject Area:	Chemical Engineering
More specific subject area:	*Flow visualization and quantification*
Method name:	*Particle tracking velocimetry for electrokinetic flows*
Name and reference of original method:	*J.C. de Valenca, R.M. Wagterveld, R.G.H. Lammertink, P.A. Tsai, Dynamics of* *microvortices induced by ion concentration polarization, Physical review. E,* *Statistical, nonlinear, and soft matter physics 92 (2015) 31003.* *A. Warren, A. Sharma, D. Zhang, G. Li, L.A. Archer, Structure and Dynamics of* *Electric-Field-Driven Convective Flows at the Interface between Liquid* *Electrolytes and Ion-Selective Membranes, Langmuir the ACS journal of* *surfaces and colloids 37 (2021) 5895–5901.* *G. Linz, S.B. Rauer, Y. Kuhn, S. Wennemaring, L. Siedler, S. Singh, M.* *Wessling, 3D*‐*Printed Bioreactor with Integrated Impedance Spectroscopy for* *Cell Barrier Monitoring, Adv. Mater. Technol. 6 (2021) 2100009.*
Resource availability:	https://www.lavision.de/en/

## Method details

With our setup, we aim to simultaneously conduct electrochemical experiments and record the velocity field of electroconvection (EC) that evolves in the electrolyte close to an ion-selective interface [1]. To ensure that the velocity field can fully develop, the respective compartment's aspect ratio should be larger than 2π [2]. Additionally, we aimed at quantifying gravitationally stable EC, which appears when the electrolyte's concentration gradient does not result in gravitational convection [3]. Therefore, concentration polarization needs to be enforced on the underside of the membrane. During the experiments, the velocity field is reconstructed in 3D with micro particle tracking velocimetry (µPTV), which demands optical access to the region of interest. In order to record the full extent of vortices, the compartment's height needs to be matched with the setup's resolvable focal depth. Furthermore, the compartment's height should be as close as possible to that of industrially used modules, which is in the order of 0.5 – 1 mm. This requirement turned out to be the key challenge during the design of the module as it introduced difficulties sealing the module and keeping the membrane from deflecting, which significantly changes the compartment's height and, thus, its electrochemical response. Another requirement derived from this is the need to record a plan view of the bottom compartment.

**Figure 1:**
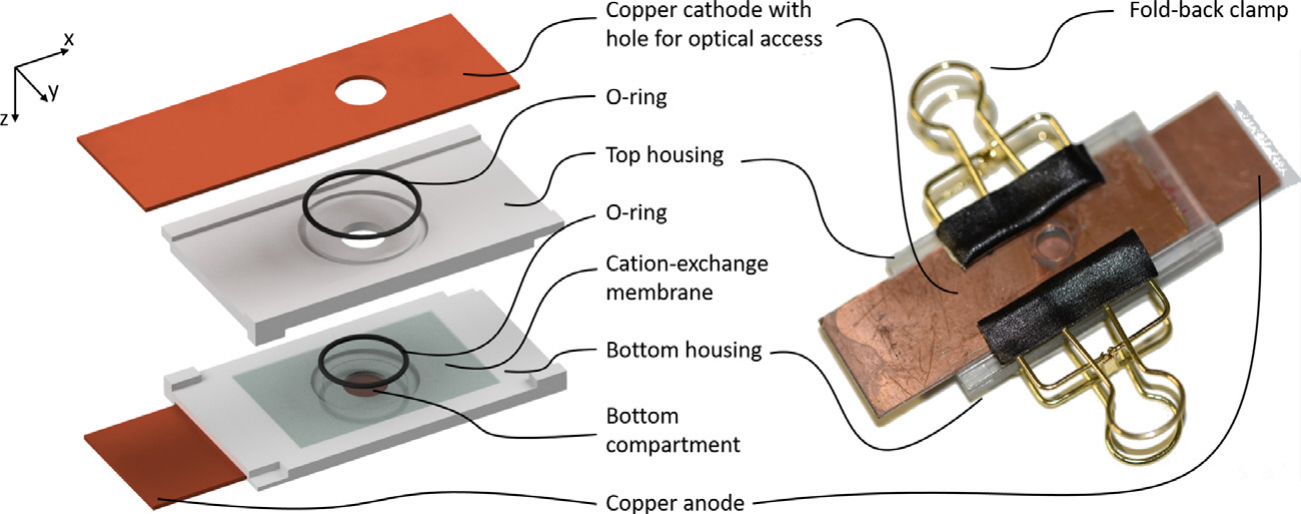
Electrochemical cell with optical access for µPTV recording. The left side shows an exploded view rendering of all parts, and the right side displays a picture of the assembled cell. A Nafion N117 cation-exchange membrane is sealed between two 3D-printed housing parts. Optical access is achieved by a drilled hole of 9 mm in the top electrode covered with a glass slide. Two fold-back clamps exert the necessary pressure to seal the assembled cell. Reprinted from Journal of Membrane Science, 640, Stockmeier et al., “Direct 3D observation and unraveling of electroconvection phenomena during concentration polarization at ion-exchange membranes”, 119846, Copyright (2021), with permission from Elsevier.

We designed the millimeter-scale electrochemical cell shown in Figure 1 to comply with all these requirements inspired by the design used in Linz et al. [4]. This design fulfills all the above-mentioned requirements allowing for the desired stable and repeatable quantification of the velocity field of electroconvection.

**Figure 2:**
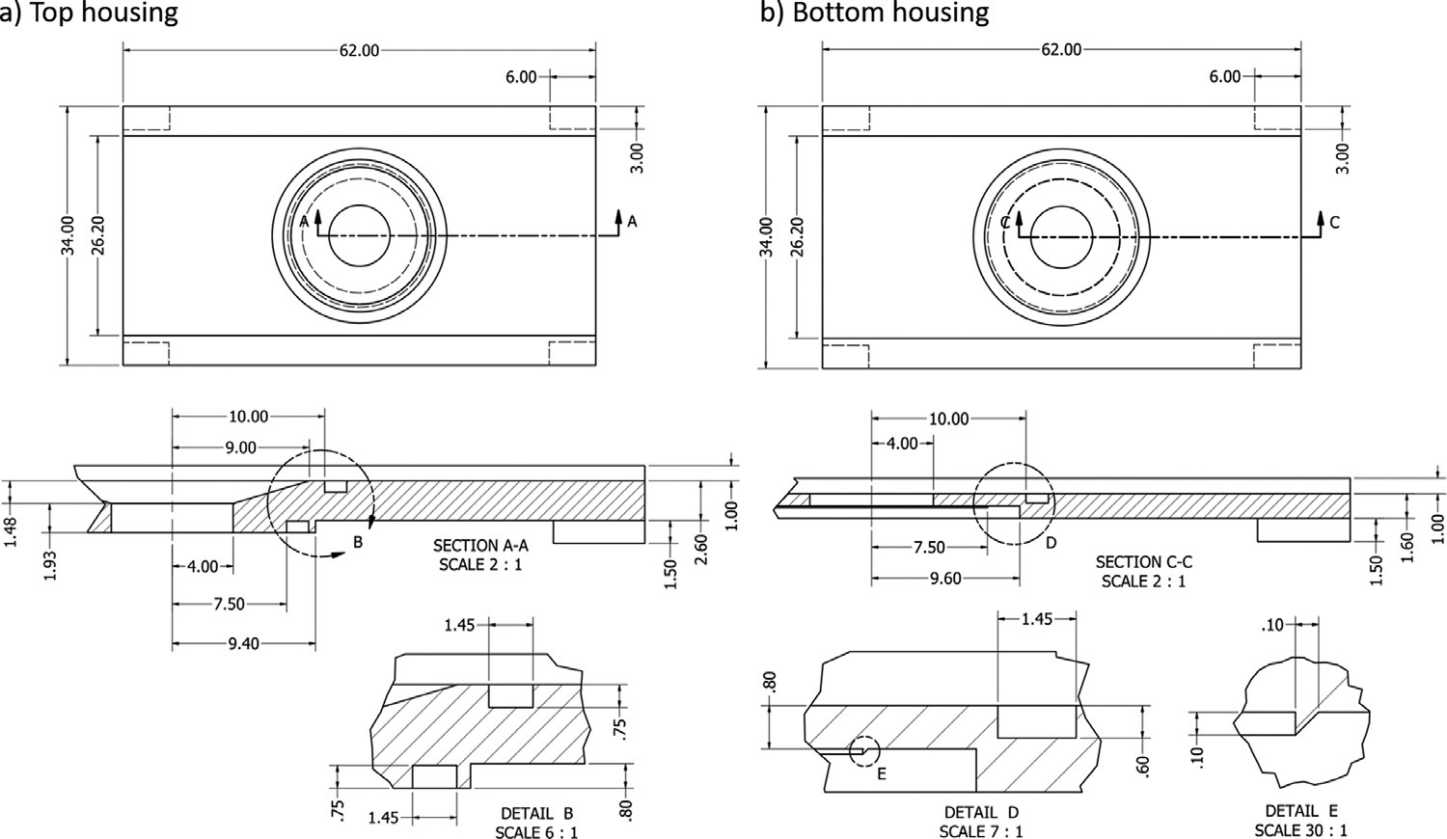
Detailed CAD drawing of the a) top and b) bottom housing part. Cross sections of the parts are shown in Sections A-A and C-C. Details B and D depict the dimensions of the O-ring grooves. Detail E highlights the sealing ridge. Dimensions are given in millimeters.

The cell's core is a Nafion N117 cation-exchange membrane sealed with an O-Ring between the two 3D printed housing parts [1]. The membrane is transparent in aqueous electrolytes, which allows for imaging through the membrane. We use two copper plates 25 mm × 75 mm × 0.5 mm as electrodes, which seal the cell's top and bottom. The cathode allows optical access through a circular hole (d = 9 mm). The hole is sealed by gluing a standard microscope glass slide (25 mm × 75 mm × 1 mm) on one side. Depending on the desired magnification, the hole's diameter can be adjusted. However, a too-small hole results in a limited field of view by cropping the light cone exiting the microscope. Both housing parts, shown in Figure 2, feature a slot with a width of 26.2 mm to fit the electrodes, centering pins and grooves at the corners, grooves for O-rings, and a central cylindrical compartment with a diameter of 8 mm, which also defines the effective membrane diameter. This diameter turned out to deliver the most reliable call assembly without out-of-scope undulation of the membrane. The top compartment, see Figure 2a), has a height of 3.41 mm and the cylinder widens to a diameter of 18 mm towards the top. The larger diameter increases both the volume and the effective electrode area. For our experiments, the bottom compartment, see Figure 2b) and also marked in Figure 1, has a membrane-to-electrode distance of 0.8 mm, resulting in an aspect ratio of 10 in relation to the membrane diameter to allow for unconstrained vortex formation. This membrane-to-electrode distance is commonly found in industrial electrochemical modules and, by design, matches the maximum focal depth of the microscope at a magnification of 5.12 × . Additionally, a large aspect ratio is desired to prevent the confinement of evolving vortices during electroconvection (EC) [5,6]. Reducing the height of the bottom compartment to 0.8 mm, was the major challenge during the cell design. It was achieved using a sharp sealing ridge, see Figure 2b) detail E, in place of an O-ring to seal the bottom compartment. In this location, the membrane is pressed against the equilateral triangle ridge with a side length of 0.1 mm by the top compartment's O-ring. Another decisive feature is the large volume and diameter ratios between the top and bottom compartments. This difference ensures more stable electrochemical conditions and a homogeneous electric field inspired by the design of De Valenca et al. [7]. Furthermore, Table 1 lists all used instruments, materials, and chemicals.

**Table 1:** Used instruments, software, materials, and chemicals.

	**Description**	**Manufacturer**
**Instruments**		
PIV system + timing unit	FlowMaster High-Speed Stereo-μPIV with PTU X	LaVision GmbH
• Cameras	Phantom VEO710L	Vision Research, Ametek, Inc.
• Fluorescence illumination	Lumen 200	Prior Scientific Instruments GmbH
• Lasers	DM150-532	Photonics Industries Inc.
• Microscope	SteREO Discovery.V20	Carl Zeiss Microscopy GmbH
• Objective lens	Plan Apo S 1.0x	Carl Zeiss Microscopy GmbH
Potentiostat	Interface 1010E	Gamry Instruments Inc.
3D-printer	Objet Eden260V/VS	Stratasys Ltd.
**Software**		
DaVis	Version: 10.0.5.47779	LaVision GmbH
MATLAB	Version: R2019b	The MathWorks Inc.
**Materials**		
3D-printing material	VeroClear RGD810	Stratasys Ltd.
Glue	UHU Plus Schnellfest	UHU GmbH & Co KG
ITO-glas	ITO-coated slide, 15-25Ω/sq	Sigma-Aldrich Chemie GmbH
Cation-exchange membrane	Nafion 117	The Chemours Company
Chemicals		
Metal salt	Copper sulfate CuSO_4_	Carl Roth GmbH & Co. KG
Tracer particles	Fluoro-Max Polystyrene Red Fluorescent 3.2μm	Thermo Fisher Scientific Inc.

For our experiments on electroconvection, we used the well-studied and straightforward electrochemical system of CuSO_4_ in an aqueous solution as the electrolyte in combination with copper electrodes and a cation-exchange membrane [1,^8^,9]. When a potential is applied, a flow of copper ions is established from the anode to the cathode. Since this system favors the deposition and dissolution of copper ($Cu^{2+}+2\;e^{-}\;\Leftrightarrow \;Cu)$, other reactions are limited and only minimum gas evolution takes place [10].

## Alteration of the setup

The electrodes, electrolyte, and membrane can be exchanged depending on the desired electrochemical phenomenon and system. However, choosing a reaction system that does not result in a significant gas bubble formation is strongly recommended. Gas evolution would obstruct the illumination and recording of the tracer particles. For example, we successfully exchanged the cathode for an ITO-coated glass slide, which allows the usage of different electrolytes without interfering with the electrochemistry of the system. When using CuSO_4_ as the electrolyte and an ITO-cathode, the resulting velocity fields were similar to those obtained with the above-described copper cathode. One downside of using ITO glass is the deposition of an opaque copper layer during experiments, which prevents extended experimental time scales.

It is possible to test other kinds of membranes and configurations under certain circumstances. The use of an anion-exchange membrane would facilitate water dissociation to gaseous products. These gas bubbles would interfere with the optical tracer recording. Additionally, the use of a different electrolyte in combination with inert electrodes, like NaCl as electrolyte with platinum electrodes, will lead to similar issues. Here, the faradaic reactions at the electrodes will produce gas bubbles. However, if the appearance of gas bubbles is limited or if the cell is oriented such that appearing bubbles will not hinder the particle recording, other membranes and electrolytes can be used in the measurements. If an opaque membrane is used, only the hydrodynamics in the chamber with optical access can be recorded. In such a case, the cell and microscope orientation need to be adjusted. If the recording of gravitationally stable electroconvection is desired, recording the bottom chamber from below the cell is possible.

Furthermore, the cell and microscope can also be oriented vertically by turning both by 90 degrees. Thereby, the gravitational situation which is most common in electrodialysis applications can be analyzed.

## Electrochemical cell validation

The assumption that an electrode with a drilled hole and introduction of tracer particles does not significantly alter the physics of the system was verified by running electrochemical experiments with three different electrode types: A copper sheet electrode as the reference, an indium tin oxide (ITO) coated glass slide as a transparent homogeneous alternative, and the copper sheet electrode with a drilled hole for optical access.

**Figure 3:**
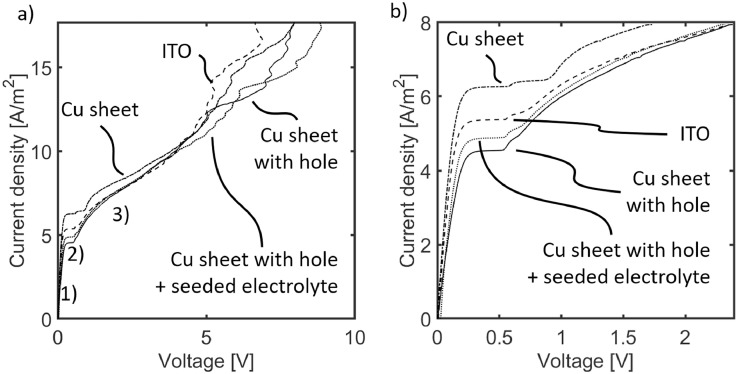
Current density over potential graph for different top electrode types and a case with a particle seeded electrolyte from Linear Sweep Amperometry experiments. The different electrode types are: Copper sheet, indium tin oxide (ITO) coated glass, and copper sheet with a drilled hole, as depicted in Figure 1. a) shows the transition from the ohmic region (1) through the plateau region (2) to the overlimiting region (3). The curves are smoothed in the overlimiting current regime. b) displays a zoom on the plateau region. The calculated limiting current density for this setup is 2.9 A/m^2^. The experiments were conducted using a 10mM CuSO_4_ aqueous solution as the electrolyte and with a scan rate of 2µA/s.

Figure 3 compares current density over potential plots for these different types of electrodes. All graphs show the typical linear relation until about 0.25 V. Thereafter, the ion transport becomes diffusion and migration limited, demonstrated by a plateau region of constant current density with increasing potential, the so-called limiting current density. With further increasing potential, the current density also increases again. The reason for the renewed close to linear increase is the formation of the electroconvective vortex field. All graphs overlap before and after the plateau region but differ in the limiting current density and plateau length. Other differences appear at large overlimiting currents. We attribute the latter to the general chaotic behavior at these current densities. The deviations in the plateau region most probably originate from the decreased conductivity in the case of ITO and the decreased electrode area in the case of the Cu sheet with a drilled hole. Still, the graphs overlap in the overlimiting region proving a similar evolution of EC. The addition of tracer particles to the system (see Figure 3: Cu sheet with hole + seeded electrolyte) leads to no significant changes.

## Velocity recording during electrochemical experiments using micro particle image velocimetry

**Figure 4:**
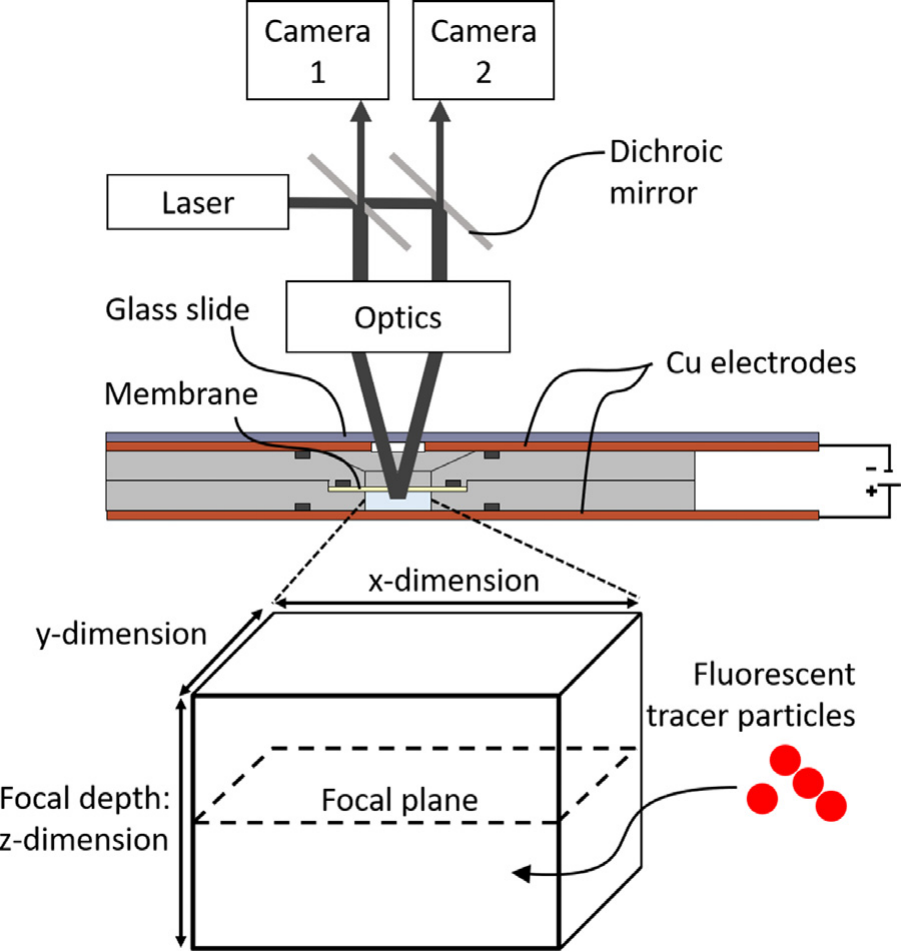
The particle tracks are recorded in a 3D volume inside the electrochemical chip via micro particle tracking velocimetry (µPTV). A stereo microscope is focused in the bottom chamber, particles are illuminated with a laser, and the tracks are recorded by two high-speed cameras. Reprinted from Journal of Membrane Science, 640, Stockmeier et al., “Direct 3D observation and unraveling of electroconvection phenomena during concentration polarization at ion-exchange membranes”, 119846, Copyright (2021), with permission from Elsevier.

During such electrochemical experiments, the electroconvective velocity field is recorded and quantified on a millimeter-scale using an optical measuring technique called micro particle tracking velocimetry (µPTV) [1]. Inert, fluorescent polystyrene microspheres (diameter: 3.2 µm; Zeta potential: -14.9 mV, Malvern Zetasizer Nano ZS; concentration: 0.001 wt. %) are used to seed the electrolyte in the anode chamber. Figure 4 illustrates the experimental setup, which includes a high-frequency, frequency-doubled 532 nm Nd:YAG laser, two high-speed cameras, and a stereo microscope with a long-distance 1 × objective. To achieve the critical measurement volume of 4.9 mm × 3.1 mm × 0.8 mm with 1280 px × 800 px resolution in x- and y-direction, which matches the depth of 0.8 mm of our electrochemical cell's anode chamber, it was necessary to halfway-close the aperture of the microscope at a magnification of 5.12 × . Here, the magnification is determined by the tracer particle diameter, so that each particle illuminates about 3 pixels in the camera images. The matching focal depth and chamber height allowed to reconstruct the electroconvective vortex field over the whole distance from membrane to anode.

The first step of the experiment is the calibration of the µPTV setup. In experiments with a halfway closed aperture, it has turned out to be indispensable to perform this calibration with laser illumination and the desired aperture setting. For this purpose, the lowest possible laser power was used.

Before usage, the ions countering the membrane's charge need to be exchanged by storing the membrane pieces in the desired electrolyte for 24h. For optimal exchange, the electrolyte needs to be renewed if the pH decreases due to the exchange of H^+^ ions from the membrane material with the desired cation. Afterwards, the membrane needs to be swollen by heating the electrolyte to 75°C. The module is assembled with the swollen membrane, clamping the membrane with the O-Ring. This ensures a flat surface when the membrane shrinks back at room temperature. Due to the elasticity of the material, the membrane surface stretches during this process.

The solution is applied directly to the membrane in the form of a large drop using a syringe. The electrode is then pressed on top so that excess solution escapes at the side, but no bubbles remain. This process is repeated for the second compartment. It is important that the two halves of the module always remain pressed together. This method has proven effective for the bubble-free filling of the chip.

After filling, the experiment needs to be quickly started to reduce sedimentation of tracer particles. Even though sedimentation is slow, it is still significant due to the high magnification and small height of the chamber. To ensure reproducible experiments, the electrodes are connected to the potentiostat, before fixing the module in place in an appropriate holder under the microscope. Thereafter, the precise channel depth, which can slightly vary in the range of micrometers between experiments, is measured by focusing the microscope at the electrode and membrane at the highest magnification and open aperture. First, the lower electrode is focused and then the underside of the membrane, which can best be identified by adhering particles. If the channel depth varies more than 50 µm from the desired 800 µm, the module needs to be reassembled using a new membrane. Before starting the experiment, it has proven useful to place the focal plane approximately 150µm below the underside of the membrane. Afterward, the calibrated magnification is set and the aperture is half-closed.

We measure the cell's resistance by electrochemical impedance spectroscopy as the real portion of the impedance at high frequencies prior to each experiment. A strong deviation in resistance is used as an indicator for extensive membrane scaling and gas bubbles (increased resistance) or cell leakage (decreased resistance). In such cases, the cell needs to be newly assembled or the membrane is exchanged for a fresh sample.

With the resistance in an acceptable range, the chonopotentiometric experiment is started at the desired overlimiting current density [1]. Depending on the goal of the experiment, the velocity field is recorded either from the start to capture the build-up of EC or after a steady-state is reached. With the specific cameras, used in his setup, the particle tracks can be recorded for a maximum of 126 s at a frequency of 100 Hz. In most cases, the recordings are oversampled and the tracks are analyzed at 20 Hz. At higher overlimiting current densities and, therefore, faster velocities, the recording frequency was set to 100 Hz. For all recordings, the optimal illumination was achieved by setting the current density of both lasers to 19 A.

## Velocity processing

**Figure 5:**
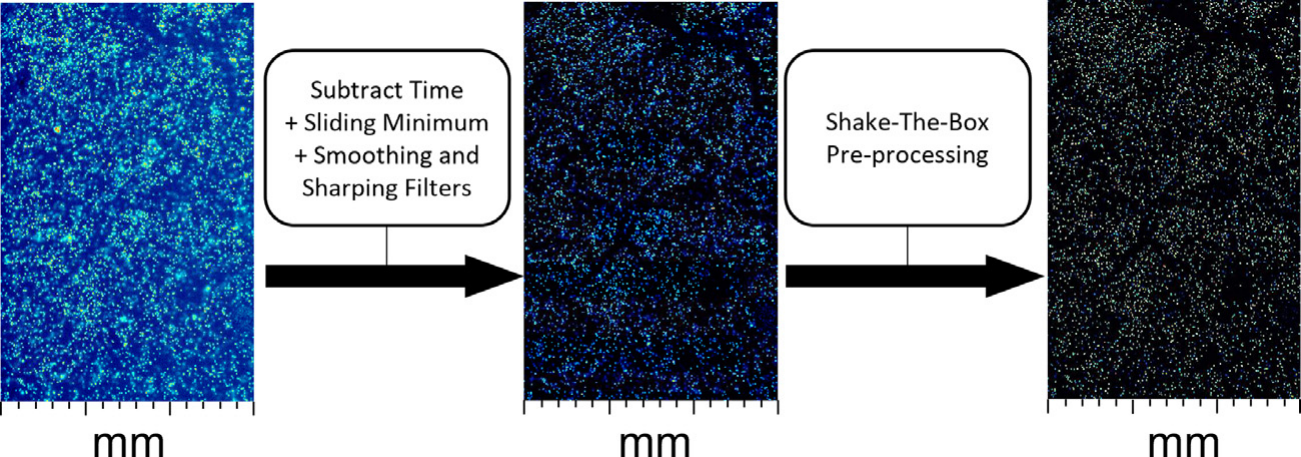
Image preprocessing.

**Figure 6:**
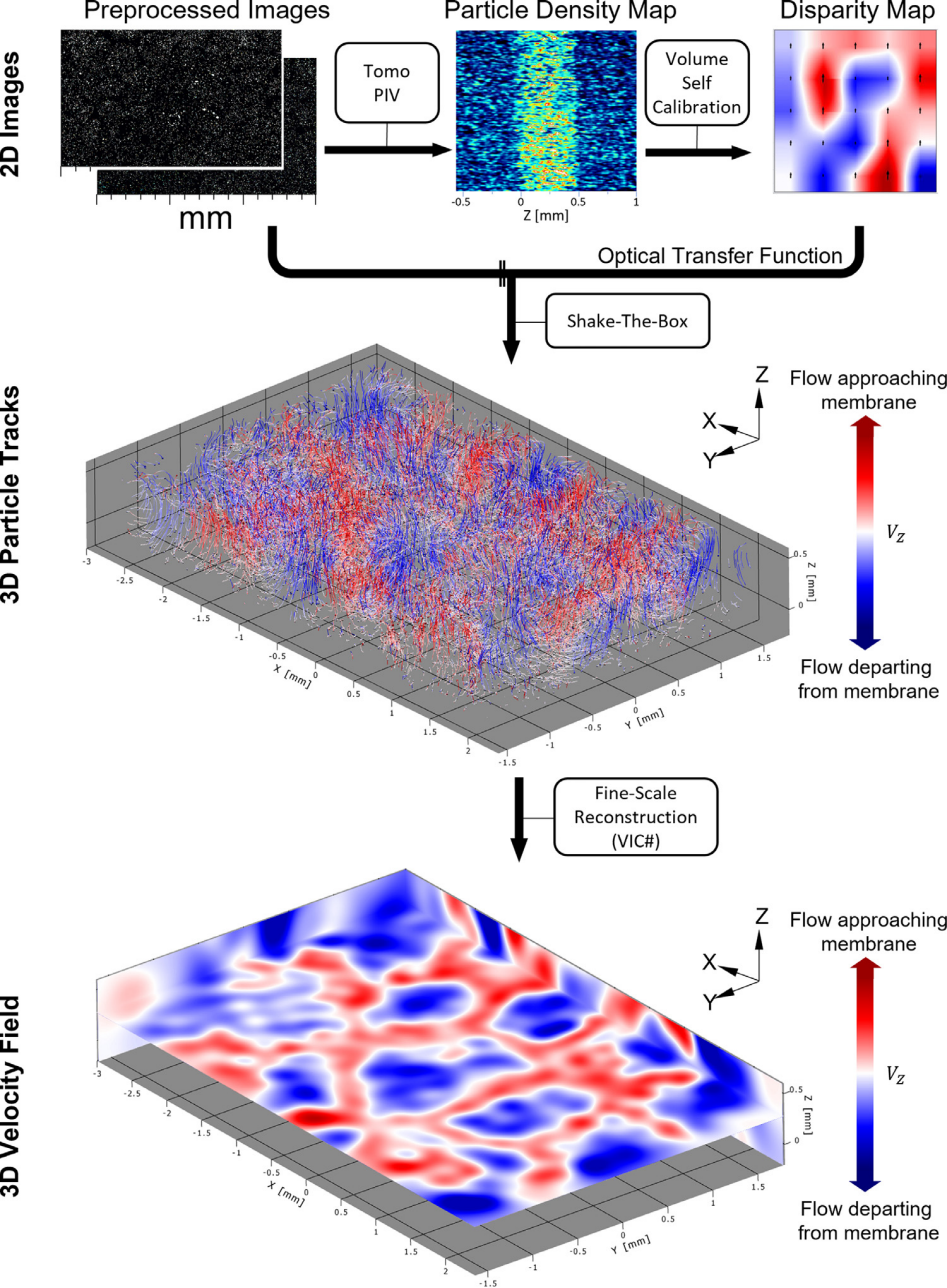
Particle reconstruction in 3D and Shake-The-Box velocity reconstruction.

After recording, the particle tracks are analyzed with the tools available in the software DaVis [1]. During processing, static particles are removed by subtracting the time-averaged intensity for each pixel. In a second step, a 3 px × 3 px sliding minimum filter together with Gaussian smoothing and sharpening is used to receive well-defined and separated particle images, see Figure 5. Subtracting 10 counts from each pixel removes the leftover background, and multiplication with a factor of two enhances the discriminability of overlapping particles. The Shake-the-Box algorithm, see Figure 6, is used to reconstruct the 3D, time-resolved particle tracks [11,12]. Due to the occasional rapid changes in particle trajectories during EC, it is necessary to increase the allowed maximum absolute change particle shift to 4 px and the maximum relative change particle shift to 100 %.

For further analysis, the particle tracks need to be converted to a velocity field on a regular grid [1]. The build-in VIC#-method is used to perform a fine scale reconstruction of the u_x_, u_y_, and u_z_ velocities [13,14]. For our recordings, reasonable computing times and accuracy, resolving all relevant features, are achieved with a grid size of 10 voxels, which yield a final resolution of 128 px × 80 px × 21 px with a voxel size of 38.4 µm.

For visualization of coherent vortex structures of EC in DaVis, the $\lambda_{2}$-method is used [1,15]. At specific eigenvalues $\lambda_{2}$, the resulting isosurfaces, which represent such coherent structures, are plotted and colored depending on their z-velocity.

The validity of the recorded velocity fields using the copper sheet electrode with a drilled hole is proven by comparing our recorded data during electroconvection to the results of a 3D direct numerical simulation in Stockmeier et al. [1]. To process the velocity field data for this comparison, it needs to be exported in DAT-format from DaVis. After importing into Matlab, the velocity data needs to be reshaped for each time step. The resulting 5D matrices (x-, y-, z-coordinate, time step, [x, y, z]-component) are non-dimensionalized by dividing by the diffusion velocity $v_{diff}=\frac{D_{CuSO_{4}}}{L_{z}}$ and used to calculate the mean square velocity which is a squared average over the x- and y-direction and in time. Additionally, the mean in x- and y-direction and time is subtracted from the velocity field for each time step and z-coordinate to calculate each voxel's fluctuating velocity component. The resulting fluctuating velocity field allows the calculation of the temporal and spatial energy spectra. These calculations are explained in detail in Stockmeier et al. [1].

## Conclusion

We believe that this method for quantifying electrohydrodynamic phenomena is a valuable tool for future studies of various electrochemical systems and processes. These include not only electrodialysis at overlimiting currents, but also hydrodynamic phenomena at gas-diffusion electrodes with the increased complexity of a three-phase system, or microfluidic systems with electrokinetic behavior.

## Declaration of competing interest

The authors declare that they have no known competing financial interests or personal relationships that could have appeared to influence the work reported in this paper.

## Data Availability

Data will be made available on request.
